# Cardiac Radionuclide Imaging in Rodents: A Review of Methods, Results, and Factors at Play

**DOI:** 10.3389/fmed.2017.00035

**Published:** 2017-03-29

**Authors:** Francesco Cicone, David Viertl, Ana Maria Quintela Pousa, Thibaut Denoël, Silvano Gnesin, Francesco Scopinaro, Marie-Catherine Vozenin, John O. Prior

**Affiliations:** ^1^Department of Nuclear Medicine and Molecular Imaging, University Hospital of Lausanne, Lausanne, Switzerland; ^2^Nuclear Medicine, Department of Surgical and Medical Sciences and Translational Medicine, “Sapienza” University of Rome, Rome, Italy; ^3^Laboratory of Radiation Oncology, Service of Radiation-Oncology, Department of Oncology, University Hospital of Lausanne, Lausanne, Switzerland; ^4^Institute of Radiation Physics, University Hospital of Lausanne, Lausanne, Switzerland

**Keywords:** small-animal imaging, rodents, myocardial scintigraphy, anesthesia, ^18^F-FDG PET, micro-PET, micro-SPECT, cardiac imaging

## Abstract

The interest around small-animal cardiac radionuclide imaging is growing as rodent models can be manipulated to allow the simulation of human diseases. In addition to new radiopharmaceuticals testing, often researchers apply well-established probes to animal models, to follow the evolution of the target disease. This reverse translation of standard radiopharmaceuticals to rodent models is complicated by technical shortcomings and by obvious differences between human and rodent cardiac physiology. In addition, radionuclide studies involving small animals are affected by several extrinsic variables, such as the choice of anesthetic. In this paper, we review the major cardiac features that can be studied with classical single-photon and positron-emitting radiopharmaceuticals, namely, cardiac function, perfusion and metabolism, as well as the results and pitfalls of small-animal radionuclide imaging techniques. In addition, we provide a concise guide to the understanding of the most frequently used anesthetics such as ketamine/xylazine, isoflurane, and pentobarbital. We address in particular their mechanisms of action and the potential effects on radionuclide imaging. Indeed, cardiac function, perfusion, and metabolism can all be significantly affected by varying anesthetics and animal handling conditions.

## Introduction

The use of small animals for radionuclide cardiac imaging is of increasing interest and goes essentially in two opposite directions. One is the reverse translation of standard protocols from humans to animals, and the other is the forward development of new probes from animals to humans. The first exploits the unique opportunity that rodents offer for simulating the evolutions of human diseases under various genetic alterations and/or pharmacological interventions, whereas the second represents a way of expanding the number of the physiopathological processes that can be exploited to produce images.

This paper is primarily aimed to be a support in the first scenario that has been outlined earlier. In particular, we wanted to show that standard radionuclide tracers are applicable to cardiac small-animal imaging at the conditions that researchers take into account and control the potential source of errors and limitations that can be encountered. We will first review the radionuclide techniques that are more often translated back to rodents, then we will give examples of the potential sources of variability that might make such translation difficult. Among these, the general characteristics of the most commonly used anesthetics in laboratory rodents will be shortly reviewed, along with their specific effects on cardiac function and additional metabolic changes potentially relevant to radionuclide cardiac studies.

## Peculiarities of Rodent Cardiac Phenotype

Several differences in cardiac anatomy and physiology exist between rodents and humans, which must be considered in translational research and that might be exacerbated in particular settings of artificially altered genetics (1). Particularly relevant to cardiac radionuclide studies are differences in cardiac dimensions, coronary anatomy, and cardiac hemodynamic. A detailed description of such differences is beyond the scope of this review as heart chambers’ volume and weight vary with age and across genders and strains. As a general rule of thumb, rodents have a heart weighting roughly three orders of magnitude less than that of humans, though heart-to-body weight ratio is similar. As far as cardiac function is concerned, heart rate (HR) is much faster in rodents than in humans, hence increasing the energetic demand. Nonetheless, stroke volume corrected for body weight is similar to humans, resulting in relatively much higher cardiac output (1). Coronary anatomy has some peculiarities as well. In mice, the interventricular septum is selectively supplied by a major septal coronary artery, while, in the absence of a circumflex artery similar to that of humans, both anterior and lateral walls of the left ventricle are perfused by the left anterior descending (2). As a consequence, the septum is always spared in ischemic models exploiting the ligation of the left anterior descending. Rats share the presence of a septal artery with mice; however, the presences of a circumflex artery and of a bigger right coronary artery make their pattern of perfusion closer to that of humans (3). Additional differences between humans and rodents pertain to the sympathetic cardiac activity. Previous studies on rats have demonstrated a higher neuronal catecholamine activity and more rapid turnover in rats than in humans (4).

## Measurement of Cardiac Function

Very high spatial and temporal resolutions are needed to cope with the small size and fast movement of the rodent heart. The first studies of cardiac function in rodents were obtained with clinical cameras or dedicated small-animal SPECT machines mounting single pinhole collimators, which allowed a spatial resolution in the range of millimeters. Newer micro-SPECT/CT cameras equipped with multi pinhole collimators offer sub-millimetrical resolution. However, detection sensitivity of SPECT and SPECT/CT cameras is generally low, requiring the injection of high activities or long scan duration. On the other hand, the resolution of micro-PET/CT systems is intrinsically limited to the range of 1–2 mm by the positron path length in tissue and by the non-collinearity of positron-electron annihilation; however, due to the lack of collimation, detection sensitivity is in general much higher with PET/CT than with SPECT/CT (5–8).

### SPECT

Left ventricular volumes and ejection fraction (LVEF) can be estimated with either gated-perfusion SPECT or gated-blood pool (GBP) SPECT with labeled erythrocytes. Due to the simultaneous visualization of both ventricular chambers, GBP allows for quantifying right ventricular volumes, as well. The feasibility and reproducibility of both perfusion and GBP SPECT was assessed by Vanhove and colleagues in healthy male Wistar rats using a clinical gamma camera equipped with a single pinhole collimator and a home-made modified reconstruction algorithm (9). By injecting 439 ± 52 MBq ^99m^Tc-sestamibi and 520 ± 49 MBq of ^99m^Tc-pertechnetate for erythrocyte labeling, respectively, these authors showed good image quality and interscan reproducibility with both techniques; however, perfusion SPECT showed better inter and intra-observer reproducibility of estimated parameters than GBP SPECT (9).

An excellent interscan reproducibility of ^99m^Tc-tetrofosmin SPECT was later confirmed by Strydhorst et al. in healthy and infarcted male Sprague-Dawley rats by means of a dedicated small-animal SPECT that allowed scaling the injected activities down to 78 ± 15 MBq (10). Though lacking direct comparison with ultrasounds or magnetic resonance (MR), this paper strongly supports longitudinal evaluation of cardiac function by perfusion SPECT, especially in models of ischemic disease, in which a larger number of animals would be required to test a single hypothesis, as intersubject variability is large (10).

A micro-SPECT camera was used by Constantinesco et al. for assessing left ventricular perfusion and function with 350–450 MBq ^99m^Tc-tetrofosmin in CD1 female mice (11). This study showed the feasibility of obtaining meaningful functional parameters, such as ventricular volumes and walls’ motion, in mice, with a resolution of about 1 mm. Interestingly, clinical available software packages (i.e., QPS and QGS, GE Healthcare) were used for the analysis after appropriate rescaling to smaller voxel size (11).

Chin and colleagues assessed left ventricular function with GBP SPECT in wild type C567BL/6 mice (^99m^Tc-pertecnetate, 444 MBq), showing GBP SPECT to be able to correctly discriminate between normal and infarcted animals, identifying segmental anomalies in this latter group (12).

More recently, other groups have used newer small-animal SPECT/CT cameras for longitudinal monitoring of mouse cardiac function after focal external beam irradiation. Perfusion images with ^99m^Tc-tetrofosmin were obtained at several time points (range of administered activities: 65–70 MBq) and functional changes were similar to that seen with ultrasounds (13, 14).

### PET

^18^Fluorodeoxyglucose (^18^F-FDG) was used as a tracer in first gated-PET studies aiming at quantifying systolic function and left ventricular volumes. Generally, only dedicated small-animal PET cameras were used for this purpose. Croteau et al. demonstrated the feasibility of accurate systolic function assessment in the volume range of 150–1,000 µL in normal and infarcted male Sprague-Dawley rats imaged with camera achieving a transaxial resolution of 2.1 mm (injected activity 111–185 MBq ^18^F-FDG) (15). In normal animals, a good correlation (*R*^2^ = 0.89) between PET and ultrasonographic estimation of ventricular volumes and LVEF was shown, whereas ventricular volumes measured with ultrasound showed a greater dispersion in diseased animals, resulting in only fair correlation with PET (*R*^2^ = 0.56). However, on average, there were no statistically significant differences between PET and ultrasound measurements (15). Few years later, Stegger and colleagues were able to show a good correlation between PET- and MR-based measurement of LVEF in control and infarcted C567BL/6 mice injected with about 10 MBq of ^18^F-FDG (*R*^2^ = 0.75, LVEF, mixed population) (16). However, end-systolic and end-diastolic volumes were slightly overestimated by the PET technique (16).

An interesting comparison between ^18^F-FDG and ^13^N-NH_3_ in healthy and infarcted male Wistar rats was later performed by Szymanski and colleagues showing a constant overestimation of ventricular volumes with ^13^N-NH_3_, which led to a significant underestimation of ejection fraction in rats with myocardial infarction (17).

Table 1 summarizes the functional parameters obtained in the aforementioned SPECT and PET seminal studies (9–12, 15–17).

**Table 1:** **Quantification of functional parameters in small-animal cardiac radionuclide studies**.

Reference	Type/sex	Strain	Technique	Presence of infarct (Y/N)	Ejection fraction (mean) (%)	End-diastolic volume (mean) (µL)	End-systolic volume (mean) (µL)
Vanhove et al. (9)	Rat/male	Wistar	^99m^Tc-Sestamibi	N	74	704	182
			Blood pool SPECT	N	65	644	227
Strydhorst et al. (10)	Rat/male	Sprague-Dawley	^99m^Tc-Tetrofosmin	N	57	470	203
				Y	45	834	462
Constantinesco et al. (11)	Mouse/female	CD1	^99m^Tc-Tetrofosmin	N	60	50	20
Chin et al. (12)	Mouse/NS	C57BL/6	Blood pool SPECT	N	64	49.9	18.1
				Y	32	73.7	53.9
Croteau et al. (15)	Rat/male	Sprague-Dawley	^18^F-FDG	N	83.2	496	90
				Y	54.6	730	353
Stegger et al. (16)	Mouse/NS	C57BL/6	^18^F-FDG	N	68	72	23
				Y	32	132	92
Szymanski et al. (17)	Rat/male	Wistar	^18^F-FDG	N	74.9	490	120
				Y	54.9	700	320
			^13^N-NH_3_	N	75.3	530	140
				Y	45.6	750	400

*The results of ancillary feasibility studies using SPECT and PET are summarized*.

## Measurement of Cardiac Perfusion

### SPECT

Radionuclide SPECT perfusion imaging of small animals relies mostly on the widely available technetium-labeled tracers, although imaging of ischemic and hypertensive rats with ^201^Tl-choride has also been described (18–20). One study suggested that image quality is superior with ^99m^Tc-sestamibi than with ^99m^Tc-tetrofosmin (21).

The feasibility of measuring the ischemic heart size with ^99m^Tc-sestamibi was tested in male Sprague-Dawley and Wistar rats by using clinical SPECT cameras, either stationary or rotating, equipped with pinhole collimators (22–24). Correlation coefficients between histology-proved and SPECT-derived infarct sizes were excellent (*R*^2^ ranging from 0.89 to 0.98), whereas defect sizes, thresholds to define a significant uptake defect on SPECT images (range: 24–70% of the normally perfused segments), and injected activities (range: 37–555 MBq) were different between studies (22–24).

A perfusion database of healthy male Sprague-Dawley rats imaged with ^99m^Tc-tetrofosmin was created in the aforementioned paper of Strydhorst and colleagues (10).

Intersubject reproducibility of perfusion SPECT was good for healthy rats and slightly poorer for infarcted animals. Interestingly, these authors reported an unexpected relatively low perfusion in the distal segments of the lateral wall (10). There is no mention to such feature in a later study on the optimization of injected activity and reconstruction parameters in male Wistar rats imaged with ^99m^Tc-tetrofosmin using cadmium–zinc telluride detectors (25).

Images of myocardial perfusion of good quality have also been obtained in mice. Wu and colleagues compared ^99m^Tc-sestamibi SPECT images obtained by means of a pinhole-equipped clinical camera with ^99m^Tc-sestamibi autoradiography in female BALB/c mice (26). A fair correlation between the two techniques was obtained (*R*^2^ = 0.72), with SPECT imaging missing some small infarcts close to the apex (26). A better correlation between ^99m^Tc-sestamibi SPECT and histological gold standard (*R*^2^ = 0.87) was found in another study using a triple-headed clinical camera equipped with single pinhole collimators on C57BL/6 male mice (27). The same amount of radioactivity was injected in these two latter studies (i.e., 370 MBq), which differed regarding the uptake thresholds for the definition of segmental defects, namely, 50 and 60% below the uptake of control groups, respectively (26, 27). A pool of normal CD1 female mice was also studied with ^99m^Tc-tetrofosmin on a dedicated small-animal SPECT, confirming a relatively lower uptake in the apex (11).

### PET

The main advantage of PET imaging is that it allows quantifying myocardial blood flow (MBF). PET radionuclide studies of cardiac perfusion are preferentially accomplished with tracers that are extracted from the blood pool with the highest efficiency, such as ^15^O-water or ^13^N-NH_3_ (28). Radiolabeled acetic acid (^11^C-acetate) has also been used as a perfusion tracer as it allows measuring the myocardial oxygen consumption (29).

Despite the most recent technological advancements and the superior quantitative ability of PET with respect to SPECT, a true kinetic modeling and quantification of MBF still represent a challenge in rodents because of small cardiac size, activity spillover, and motion artifacts, which produce a significant partial volume effect. The impracticability of repeated blood sampling is an additional obstacle, especially in mice, and image-derived input functions from heart ventricles suffer from significant errors, exceeding 100% in rats and 400% in mice (30, 31). Therefore, alternative models for input function quantification have been proposed (30). By contrast, quantification errors due to self-absorption (attenuation) of radioactivity are less relevant, and attenuation correction is rarely taken into account in small-animal imaging studies. However, recent literature has suggested that photon attenuation should be corrected for especially in studies in which animals of different body weights are compared (32). Moreover, as will be detailed below, the type of anesthesia can significantly influence the quantification of MBF.

A number of studies have shown the feasibility and repeatability of PET measurements of MBF in non-ischemic rats. Reported MBF varies between 3 and 6 mL/min/g, is age related, and compares well with reference measurements obtained by radioactive microspheres or MR (29, 33–35). As it will become clear later in this review, these measurements are largely dependent on the type and conditions of anesthesia.

In male Sprague-Dawley rats, Kudo and colleagues obtained a good linear correlation (*R*^2^ = 0.86) between the extent of ^13^N-NH_3_ uptake defect—defined as showing <50% of the highest uptake—and the fraction of infarcted myocardium (33). The same group exploited the use of ^13^N-NH_3_ for quantifying the modifications of myocardial perfusion in C57BL/6 mice induced by the negative chronotropic effect of the α-2 agonist clonidine. By using a ROI-based approach, a 14% reduction of ^13^N-NH_3_ uptake was demonstrated after 50% depression of HR induced by clonidine. Owing to the flow-dependent myocardial tracer kinetics, the 14% reduction in ^13^N-NH_3_ uptake would correspond to a 50% reduction of cardiac blood flow, although this was only an indirect estimation (36).

Several fluorinated PET perfusion tracers, such as ^18^F-flurpiridaz, with mechanism of uptake similar to that of classical SPECT tracers ^99^Tc-tetrofosmin and ^99^Tc-sestamibi, have been recently tested in small animals and are under evaluation at a clinical level. These have been recently reviewed (37). Finally, the first ^82^Rb PET image of a Sprague-Dawley rat with myocardial infarct has been recently released and shows that imaging with this tracer is feasible (38). It remains to be proven, however, that the high energetic ^82^Rb positron emission, with consequent longer path length in tissue, does not negatively affect image quality, especially in mice.

## Measurement of Cardiac Metabolism

In normal conditions, cardiac energetic production relies primarily on fatty acid metabolism (70–90%), whereas glycolysis plays a secondary role (10–30%). The hibernating myocardium, namely, a portion of viable heart that survived an acute ischemia or a chronic hypoperfusion, is characterized by a glycolytic switch together with reactivation of the fetal gene program (39). Imaging of the viable myocardium with ^18^F-FDG is a well-established clinical procedure used to select suitable candidates for coronary revascularization.

Cardiac hypertrophy is also characterized by a glycolytic switch and attracts the attention of the researchers studying the natural history of the disease, potential pharmacological interventions, and genetic or environmental risk factors. As a consequence, a number of studies have used ^18^F-FDG in combination with radiolabeled fatty acid analogs in the assessment of rats with spontaneous or induced left ventricular hypertrophy, confirming the expected trend of increasing ^18^F-FDG uptake with progressing disease (40–43).

Less clear are the modifications undergone by fatty acid metabolism during the course of the disease which, together with the complexity of the involved metabolic pathways, might explain the absence of a widespread and standardized clinical use of radiolabeled fatty acid analogs in this and other cardiac conditions (44).

^18^F-FDG PET has very favorable physical properties and broad availability, appearing as a simple and robust imaging modality to assess a number of pathological conditions and their functional and metabolic consequences. Thus, due to prominent cardiac uptake in basal conditions, triggered by uncontrolled dietary status and external factors such as the use of anesthetics, ^18^F-FDG is frequently used to assess cardiac perfusion after an ischemic insult in rodent models.

In outbred wild-type mice, Stegger and colleagues found an excellent linear correlation (*R*^2^ = 0.96) between ^18^F-FDG uptake defects (i.e., <50% of the highest myocardial uptake) and the histological gold standard after permanent coronary ligation (45). Similar results were later confirmed by Higuchi and colleagues in Wistar rats (46). However, because of the unmet need of very high resolution, these analyses were limited to transmural infarcts.

The question remains as to whether ^18^F-FDG PET/CT is a suitable modality for quantification of non-transmural infarct in rodents. ^18^F-FDG was also tested against histology and compared with other imaging modalities in models of acute reperfused myocardial infarct in male Sprague-Dawley rats. Two independent groups showed a mismatch between FDG uptake and that of perfusion probes (^13^N-NH_3_ and ^99m^Tc-sestamibi, respectively), indicating the ability of FDG to detect peri-infarcted areas of stunned myocardium with a glycolytic switch (47, 48). McNulty and colleagues very elegantly showed such images of increased FDG uptake to correspond to “no-reflow” zones with concomitant transient glycogen depletion (47).

Dietary changes significantly affect the ^18^F-FDG distribution in organs with inducible glucose transporters such as striated and cardiac muscle. On the other hand, tissues with constitutive, non-insulin-dependent glucose metabolism such as the brain do not change their rate of glucose consumption according to the dietary state.

A full analogy between glucose and ^18^F-FDG metabolism, expressed by a lumped constant (LC) equal to 1, is generally considered in cardiac human studies, even during euglycemic insulin infusion (49). A constant LC might not apply to ischemic hearts, simplified artificial systems or to different animal species (50).

The dependence of cardiac ^18^F-FDG uptake from blood glucose and insulin levels is not straightforward, as additional factors such as lipid pool are of significant impact. As a general rule, fasting reduces blood glucose and insulin levels, thereby increasing relative fatty acid metabolism and reducing cardiac ^18^F-FDG uptake. By contrast, administration of glucose and/or insulin significantly increases cardiac glucose metabolism and, consequently, ^18^F-FDG uptake. Notably, the pattern of cardiac ^18^F-FDG uptake under hyperglycemia might be biphasic, showing a paradoxical inhibition with glucose blood levels above 11.1 mmol/L (200 mg/dL), as elegantly demonstrated by Kubota and colleagues in rats (51).

Kreissl and colleagues studied the effects of fasting and insulin administration in C57BL/6 mice by keeping the conditions of anesthesia stable (i.e., isoflurane 2% in 100% oxygen) (52). As expected, the fasting overnight significantly reduced plasma glucose levels and cardiac ^18^F-FDG uptake. Accordingly, kinetic analysis showed significantly reduced ^18^F-FDG uptake rate constant (*K*_i_) and glucose metabolic rates (MR_glu_) in the fasting conditions compared to fed animals. Insulin administration increased *K*_i_ in all animals; ^18^F-FDG uptake was increased in fasting animals, whereas no significant changes of ^18^F-FDG uptake were seen in fed animals (52).

## Variables Affecting Cardiac Radionuclide Studies in Rodents: Types of Anesthesia

For obvious reasons, all imaging studies with rodents require general anesthesia.

### Ketamine/Xylazine

The cyclohexamine ketamine, a phencyclidine derivate, is the most widely used veterinary anesthetic in combination with α-2 adrenergic agonists such as xylazine or medetomidine (53, 54). Preferred administration route is intraperitoneal (IP), although ketamine can also be administered intravenously and intramuscularly. Common dosage in rodents is 80–200 mg/kg given intraperitoneally.

Ketamine inhibits glutamatergic postsynaptic neuronal depolarization by non-competitive binding to the phencyclidine receptor in the *N*-methyl-d-aspartate (NMDA) channel (55), producing a cataleptic state along with analgesia, immobility, dissociation from the environment, and amnesia. It does not globally reduce cortical metabolism and glutamate release, and it is therefore categorized as dissociative anesthetic. In addition, ketamine has profound central sympathomimetic effects that are responsible for the most common side effects such as hallucinations and increase of blood pressure and HR in humans. This central sympathetic stimulation, however, is partially masked by a concentration-dependent direct negative inotropic effect of ketamine on the myocardium, which is common to several mammals but controversial in rodents (56–58).

In veterinary medicine, most often ketamine is used in combination with xylazine, which confers muscle relaxation. The dose of xylazine is usually adjusted to achieve a ketamine/xylazine dose ratio of 10/0.5–1.

In rodents, the net effect of the combination ketamine/xylazine on heart function is a significant bradycardia (>50% reduction of HR), enlarged end-diastolic dimension and reduced systolic function. This has been consistently shown by cardiac catheterism as well as ultrasound and MR techniques (59–61). Notably, these effects are time and strain dependent (62) and can be reversed by α-2 receptor blockers (e.g., atipamezole) (59).

The ketamine/xylazine mixture produces prominent systemic metabolic changes, particularly in the metabolism of glucose and catecholamine. As shown by Saha and colleagues in fed male Sprague-Dawley rats, blood glucose levels increase above 8.8 mmol/L (160 mg/dL) as early as 20 min after administration of ketamine/xylazine, reaching peak levels of 16.1 ± 1.4 mmol/L (291.7 ± 26.6 mg/dL) 120 min postinjection (63). In this study, hyperglycemia (>8.8 mmol/L) was maintained during the entire length of the experiment (180 min). Fasting animals showed a less steep increase of blood glucose levels, which never rose above the hyperglycemic threshold and came back to initial values within 150 min (63). A significant rise of glucose levels, secondary to ketamine/xylazine anesthesia and dependent on the dietary status, was confirmed by Lee and colleagues in C57BL/6 tumor-bearing mice (64). According to Fueger et al., fasting has little influence on hyperglycemia induced by ketamine/xylazine in severe combined immunodeficient (SCID) mice (65). Concomitant changes are observed in glucose regulatory hormones, in particular insulin and glucagon. Indeed, plasma insulin levels drop in a time-dependent manner within the first 30 min after ketamine/xylazine administration in fed animals, and then reach a steady low level after a small peak increase 90 min postinjection. On the other hand, glucagon levels show profound increase between 60 and 180 min after ketamine/xylazine injection (63). All these effects are significantly attenuated in fasted animals that have lower baseline hormone levels and reduced glycogen stores (63, 64).

Ketamine alone does not produce any modification on either glucose levels or insulin release; therefore, the variations in glucose levels seem to be largely mediated by xylazine, which inhibits insulin release from pancreatic islet cells (66). This mechanism is also strongly supported by the counter effects on glucose and insulin levels produced by the α-2 adrenergic antagonist yohimbine, whereas a regulatory effect of α-2 receptors on glucagon levels has not been univocally proven (63).

Additional modifications with variable kinetics were also seen in fed rats regarding adrenocorticotrophic hormone and corticosteroid (i.e., inhibition) as well as in growth hormone (i.e., increase) (63). Moreover, catecholamine plasma levels and peripheral activity might fluctuate due to the effects of ketamine and xylazine, potentially interfering with radionuclide studies exploring this metabolic pathway (67).

### Isoflurane

Isoflurane is a volatile halogenated anesthetic producing unconsciousness and immobility without significant analgesia. These most relevant features of isoflurane are obtained by distinct interactions with both central and peripheral nervous system. Similar to other inhaled agents of the same class, the effect on the brain is depression of several regions, largely mediated by sensitization and prolonged response of γ-aminobutyric acid type A (GABA_A_) receptors to their ligand. Additional central inhibitory effects are exerted on several channels including the nicotinic acetylcholine and serotonin receptors. Immobilization is thought to be obtained by increased activity of inhibitory glycine receptors and inhibition of NMDA and AMPA receptors (68).

Isoflurane has a very fast onset of action, and it is rapidly washed out after withdrawal. There is only little metabolism by the liver. Cardiac effects of isoflurane result from inhibition of potassium and calcium channels, inducing negative chronotropic and inotropic actions.

In a study involving 27 mice of different strains, Szczesny and colleagues found no significant changes in mean arterial blood pressure and HR during 4 h under isoflurane anesthesia vaporized at 1–1.3% (69). A near-linear significant drop in arterial blood pressure was observed above the threshold of 2% vaporization. These mice were kept under continuous administration of isotonic saline over the entire study period to maintain fluid balance (69). In accordance with that, additional studies found no changes in HR in both rats and mice under isoflurane anesthesia (61, 70). On the other hand, different authors found a small reduction of HR and cardiac index with isoflurane, through these effects are less prominent than observed under other types of anesthesia such as ketamine/xylazine and pentobarbital (59, 62).

Interestingly, variability in cardiac function between mouse strains in response to isoflurane appears to be not significant (62, 69), except for a slightly higher HR depression found on BALB/c mice (71).

Other relevant cardiac effects of isoflurane are dose-dependent increased MBF (61, 70, 72, 73) and reduced infarct size after ischemia–reperfusion (74).

As regards glucose metabolism, in fed male Sprague-Dawley rats, Saha and colleagues observed a rapid blood glucose increase after the onset of isoflurane anesthesia, albeit less pronounced if compared to ketamine/xylazine (peak levels: 232.4 ± 26.3 mg/dL, about 150 min from baseline) (63). In fasted rats, increase in glucose levels did not reach significance (63). Zapp and colleagues confirmed hyperglycemia following isoflurane in Long–Evans rats (75). Fueger et al. had similar results in SCID mice (65). On the contrary, Dandekar et al. and Loepke et al. showed decreased glucose levels induced by isoflurane in melanoma-bearing nude mice and C57BL/6 pups, respectively (76, 77). Such different profile of glucose levels remains unexplained, though it might be attributable to differences in animal handling or mice strains.

The most severe adverse effect of isoflurane is depression of respiratory rate, superior to that induced by ketamine/xylazine and pentobarbital; however, tidal volume seems to be better maintained during isoflurane anesthesia, accounting for relatively higher peripheral capillary oxygen saturation (SPO_2_) compared with pentobarbital (78).

### Pentobarbital

Pentobarbital belongs to barbiturates, a class of GABA mimetic anesthetics, which elicit their action interacting with different GABA_A_ receptor subunits from those targeted by volatile anesthetics (79).

Barbiturates are not the anesthetic of first choice in small-animal research because of the lack of significant analgesic effects and of the narrow safety margin. However, the relative inexpensiveness, rapid onset of unconsciousness and easy of IP injection make them a valuable option. The metabolism of barbiturates occurs mainly in the liver; pentobarbital is metabolized primarily by the hepatic cytochrome P450. Large variability in pharmacokinetic and pharmacologic effect is observed between species, strains, and single individuals. Recommended dosage of pentobarbital varies between 40 and 60 mg/kg given intraperitoneally (53).

Although the wide range of dosages makes comparisons between studies difficult, observed depressive effects on cardiac function in rodents were generally less severe with pentobarbital than with ketamine/xylazine (59, 80). Interestingly, no significant effects on blood glucose levels have been observed (63); however, some authors found a twofold increase of insulin levels in C567BL/6 tumor-bearing mice injected with 50 mg/kg pentobarbital after 4 h of fasting (64). Such effect was not observed after 20 h of fasting, suggesting that it might be related to transient fluctuations of glucose levels (64). Respiratory depression with reduction of SPO_2_ is of most concern after administration of pentobarbital (78).

## Impact of Anesthesia on Cardiac Radionuclide Imaging in Rodents

The choice of anesthetic has profound effects on cardiac radionuclide imaging in rodents. Thackeray and colleagues showed no impact of fasting on blood glucose levels and cardiac ^18^F-FDG uptake in C57BL/6 mice, while differences in cardiac ^18^F-FDG distribution between study groups were largely attributable to changes in the anesthetic protocol (81). In particular, cardiac ^18^F-FDG uptake was markedly higher under continuous isoflurane anesthesia than after ketamine/xylazine (30.2 ± 7.9 vs 2.4 ± 2.2% ID/kg, respectively) (81). These authors also found that further reduction of cardiac uptake could be obtained by limiting the isoflurane anesthetic time to the length of the acquisition, by extending fasting duration to 18 h and by giving heparin pretreatment (500 U/kg intravenously) 15 min prior to ^18^F-FDG (81). Several independent research groups confirmed predominant effects of anesthesia on cardiac ^18^F-FDG uptake, indicating significantly higher values under isoflurane anesthesia than with ketamine/xylazine or other anesthetic agents (64, 65, 82–84). Besides changes in glucose metabolism, an additional possible explanation for reduced cardiac ^18^F-FDG uptake under ketamine/xylazine or pentobarbital would be the depressive effects of these agents on cardiac contractility and HR which, in contrast, are not significantly modified under isoflurane anesthesia.

**Figure 1:**
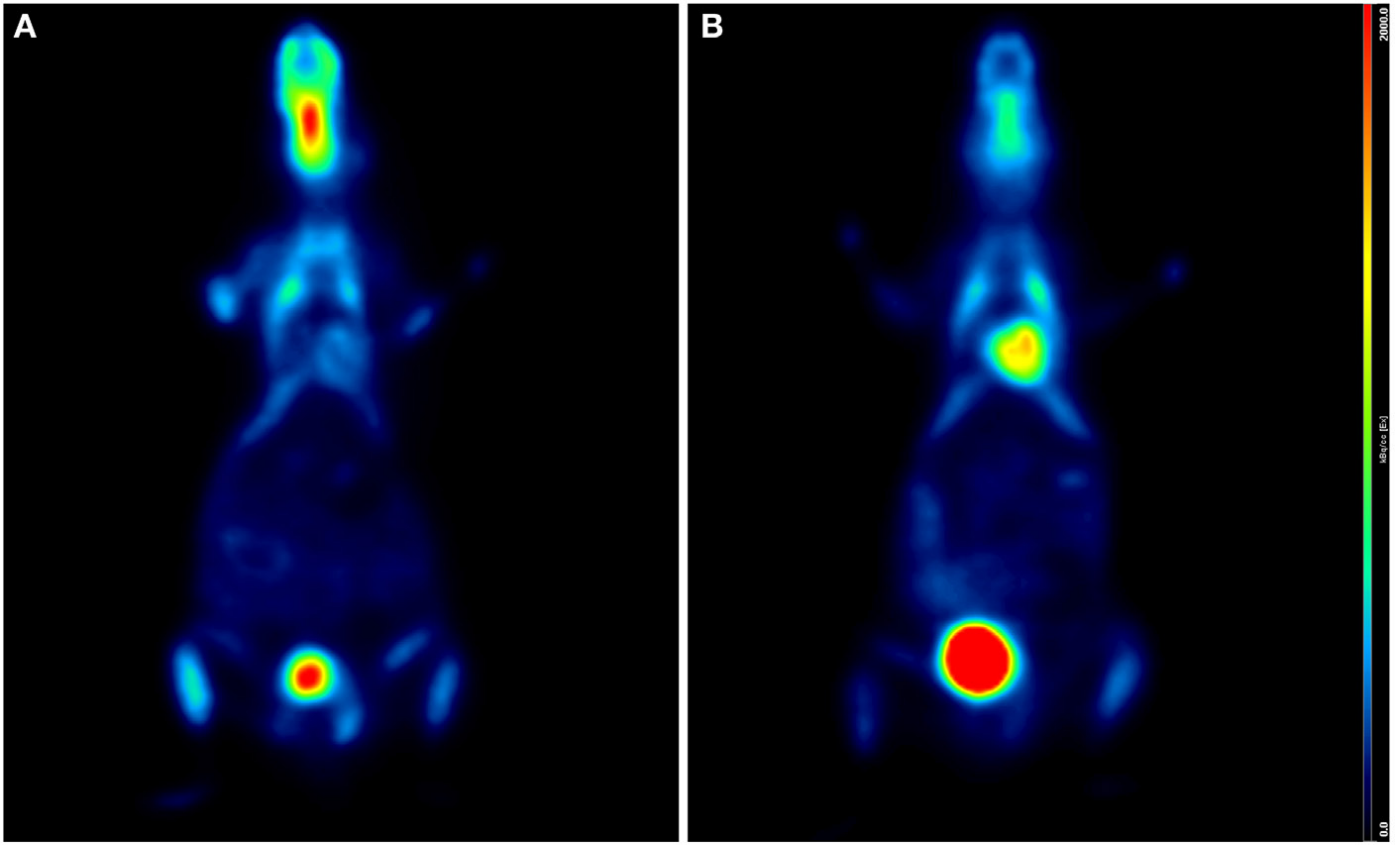
**Influence of anesthetic drug on cardiac ^18^F-FDG uptake**. Coronal PET/CT (micro-SPECT/PET/CT Albira, Bruker) slices of two age- and weight-matched C57BL/6 male mice acquired under ketamine (50 mg/kg)/medetomidine (1 mg/kg) **(A)** and isoflurane (2%, 1 L/min air) **(B)**, 60 min after intraperitoneal injection of 15 MBq ^18^F-FDG, respectively. Higher FDG uptake is clearly seen in panel **(B)**. Both animals were awake and warmed on a heating pad during the uptake phase.

**Figure 2:**
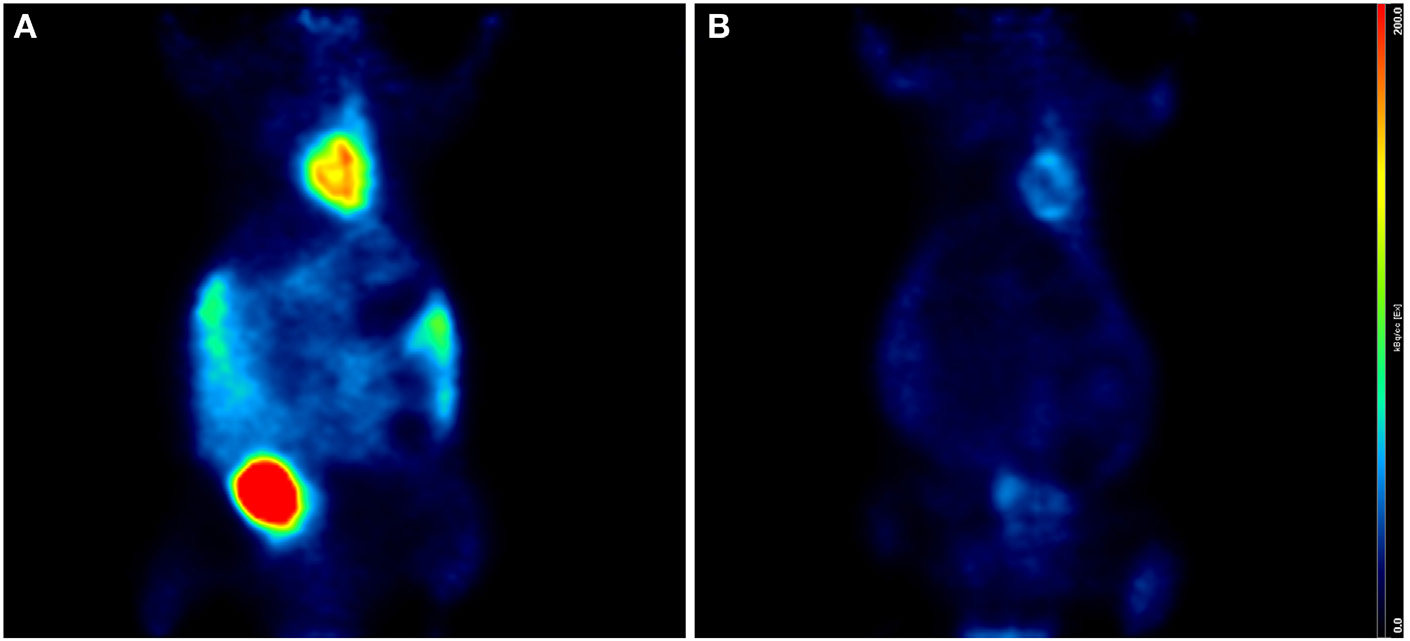
**Influence of length of anesthesia on cardiac uptake**. Coronal PET/CT (microSPECT/PET/CT Albira, Bruker) slices of two age- and weight-matched Balb/c male mice acquired 60 min after intraperitoneal injection of 15 MBq ^18^F-FDG. Both animals were warmed with a heating pad during the uptake phase; however, mouse **(A)** was kept under isoflurane anesthesia (2%, 1 L/min air) during the entire procedure, while mouse **(B)** was anesthetized only during the acquisition. Cardiac uptake is higher in mouse **(A)** than in mouse **(B)**, in keeping with the longer exposition to isoflurane.

As previously described, an additional relevant effect of anesthesia is the increased myocardial perfusion induced by isoflurane, which might significantly influence the quantification of MBF (29). Effects of different anesthetic drugs and different duration of anesthesia are shown in Figures 1 and 2, respectively.

## Other Variables Affecting Cardiac Radionuclide Studies

### Route of Injection

Several routes of injections are possible in small-animal imaging; these of course have an impact on imaging results. Intravenous (IV) is the standard route of injection; however, it suffers from unpredictable errors due to difficulties in venous cannulation and venous fragility, leading to tracer extravasations, especially in mice. A subjective qualitative evaluation might suffice for determining the goodness of the injection, although quantitative corrections should be applied when quantification of tracer uptake is mandated (85, 86). Attention must be paid to avoid bubbles and to not inject excessive volumes—with differences among sex, age, and strains—usually not larger than 5 mL/kg in mice. An additional issue might be the speed of injection for tracers with potential systemic effects, such as carrier added *meta*-iodo-benzyl-guanidine.

Other suitable routes of injection are IP and retroorbital (RO). In an *ad hoc* comparative study, IV and RO proved to be nearly identical in terms of pharmacokinetics and extent of organ ^18^F-FDG uptake (87). As expected, IP injections showed slower kinetics with residual abdominal radioactivity in some cases (87). The chance of erroneous intra-intestinal administration must be also taken into account in a non-negligible percentage of cases (10–25%) (88). As confirmed by several groups, the differences in biodistribution between IP- and IV-injected ^18^F-FDG usually vanish at later time points, namely 60 min. Therefore, IP route seems to be a practical and robust alternative to IV in case of cardiac radionuclide studies using ^18^F-FDG (65, 87, 89, 90). It is stressed, however, that the concentration of the injected radionuclide should be kept consistently the same throughout the study, as more concentrated compounds might be retained in the abdominal cavity thereby reducing the availability in the bloodstream (90). Moreover, IP injections should be validated against IV on a single agent basis, as different molecular structures might be absorbed differently.

Interestingly, the equivalence between IP and IV injections does not apparently hold true for SPECT perfusion tracers such as ^99m^Tc-sestamibi and ^99m^Tc-tetrofosmin. In the study by Vrachimis et al., image quality was much lower with IP than with IV injection for both tracers (21). Moreover, ^99m^Tc-tetrofosmin was comparable to ^99m^Tc-Sestamibi if injected RO, but not if injected IV. Best image quality for RO injections was found earlier (30 min postinjection) with ^99m^Tc-tetrofosmin than with ^99m^Tc-sestamibi (48 min postinjection) (21).

### Animal Handling

**Figure 3:**
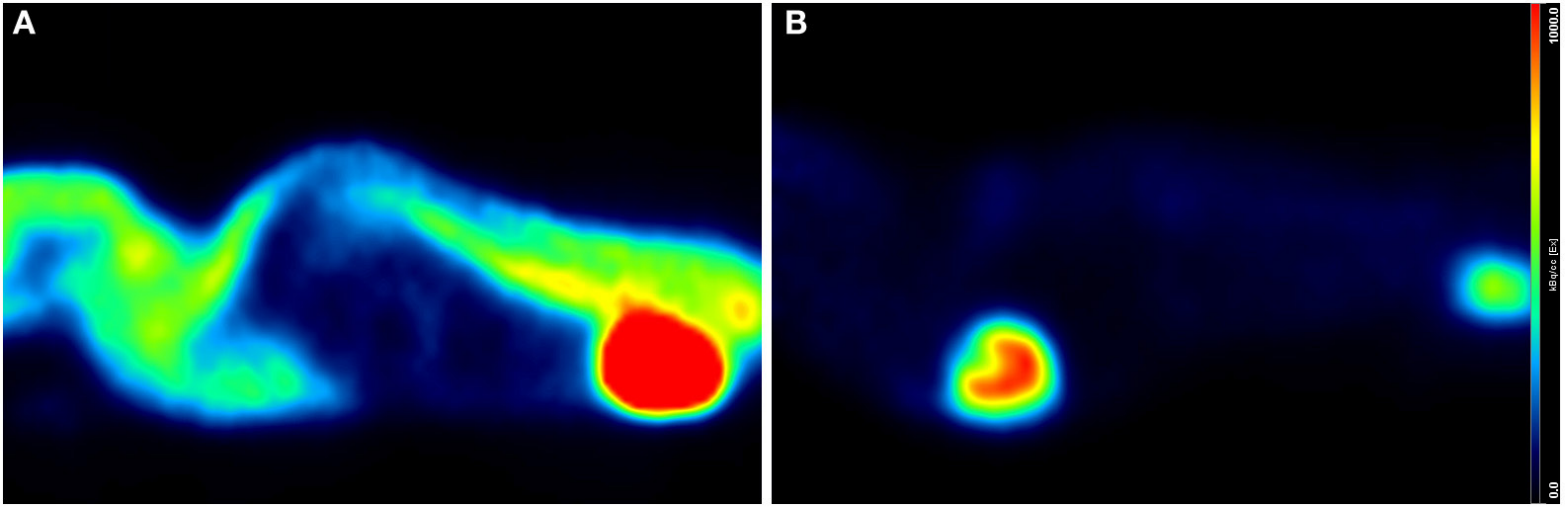
**Influence of warming on whole-body and cardiac ^18^F-FDG uptake**. Sagittal PET/CT (micro-SPECT/PET/CT Albira, Bruker) slices of two age- and weight-matched Balb/c male mice acquired under isoflurane (2%, 1 L/min air), 60 min after intravenous injection of 15 MBq ^18^F-FDG. Both mice were awake during the uptake phase but warming conditions were different: mouse **(A)** was not warmed, whereas mouse **(B)** was kept on a heating pad. Increased muscular and brown fat uptake as well as decreased cardiac activity is seen in **(A)**, while panel **(B)** shows low background and enhanced cardiac uptake.

A correct animal handling is of paramount importance for the quality of small-animal imaging. Animal body temperature may decrease by several degrees with anesthesia; therefore, it needs to be strictly monitored during the experiments (78). In fact, non-warmed animals show a high uptake by brown fat, skeletal muscle and Harderian glands, which might interfere with the quality of images and their reproducibility. Constant warming during anesthesia, significantly reduce such abnormal uptakes (65) (see Figure 3).

## Conclusion

State-of-the-art small-animal radionuclide imaging techniques allow the study of several cardiac features in rats and smaller rodents. However, some computational limitations exist especially in mice, due to animal weaknesses, small heart dimensions, and fast movement. Besides that, a number of variables, not encountered in human studies and influencing imaging results, should be taken into account and controlled, in order to ensure consistency and reproducibility of data. Among these, the conditions of anesthesia are of upmost importance either because of their direct effects on cardiac function or due to their systemic effects, interfering with whole-body and cardiac metabolism.

## Animal Care

All animal experiments were performed according to the principles of laboratory animal care and national ethical guidelines. The animal experiments have been subjected to authorization and control by the official Canton and Swiss veterinary service on surveillance of animal experiments.

## Author Contributions

Conception and design of the work: FC, DV, FS, M-CV, and JP. Data collection: FC, DV, AP, TD, and SG. Data analysis and interpretation: FC, DV, AP, TD, SG, FS, M-CV, and JP. Manuscript writing: FC and DV. Critical revision of the article: FS, M-CV, and JP. Approval of the final version of the article: FC, DV, AP, TD, SG, FS, M-CV, and JP.

## Conflict of Interest Statement

The authors declare that the research was conducted in the absence of any commercial or financial relationships that could be construed as a potential conflict of interest.
